# Pt incorporated mesoporous carbon spheres: controllable structure with enhanced catalytic activity and stability†

**DOI:** 10.1039/c8ra01453a

**Published:** 2018-04-16

**Authors:** Hongyan Liu, Yaling Liu, Huimei Da, Ruo Yuan

**Affiliations:** Key Laboratory of Luminescent and Real-Time Analytical Chemistry, Ministry of Education, College of Chemistry and Chemical Engineering, Southwest University Chongqing 400715 P. R. China liuhy860@swu.edu.cn

## Abstract

We report a simple synthesis process to prepare well-dispersed Pt nanoparticles incorporated in mesoporous carbon spheres. By manipulating the relative ratio of Pt precursor and resorcinol-formaldehyde resin (RF), Pt/carbon composites with different morphologies and Pt content were achieved. The as-prepared Pt/C composite materials show higher catalytic activity and reusability for the reduction of 4-nitrophenol (4-NP) than the Pt deposited commercial activated carbon (Pt/AC), which can be ascribed to the high dispersion of Pt nanoparticles in the carbon spheres.

## Abstract 2

We report a simple synthesis process to prepare well-dispersed Pt nanoparticles incorporated in mesoporous carbon spheres.

## Graphical abstract

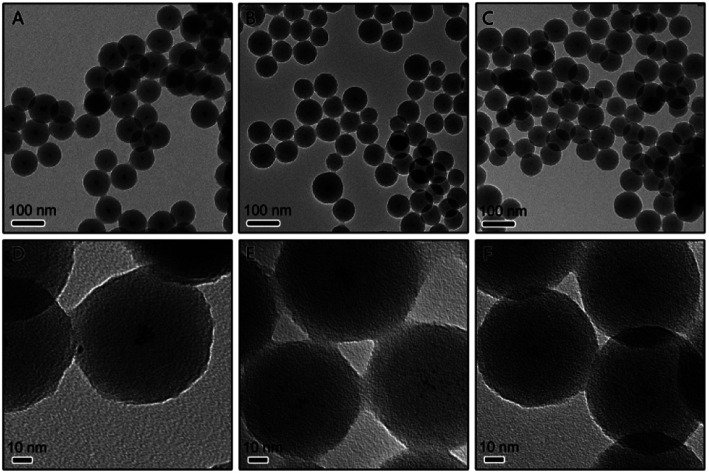

## Introduction

Noble metal nanoparticles (NPs) including Pt, Pd, and Au have attracted significant attention owing to their broad applications as catalysts in reactions such as hydrogenation, dehydrogenation and oxidation.^1–3^ However, the extremely high-cost and rare resources limit their large scale applications. Therefore, it is desirable to improve the utilization efficiency of noble metals.^4^ Many efforts have focused on improving catalytic effectiveness by dispersing noble metal catalyst onto an appropriate support with high surface area, such as metallic oxides.^5–8^ Among the various support materials, carbon materials have attracted tremendous research interest due to the attractive properties such as low cost, high surface area and excellent thermal stability.^9,10^ To date, a lot of works have been focused on decorating metal NPs on carbon-based nanomaterials including carbon fiber,^11^ carbon nanotube,^12,13^ hollow carbon sphere,^14,15^ and graphene.^16,17^ The activities of the noble metal catalysts could be significantly improved by reducing the particle size and increasing their distribution density on the support material.^18,19^ However, small-sized metal NPs tend to sinter and grow into larger particles, and that leads to the loss of the unique catalytic properties seen in the original nanoparticles.^20,21^ Therefore, effective control of metal size and dispersion in the carbon–metal composite is a critical concern in designing and synthesizing high-performance heterogeneous catalysts.

To address this short-coming, metal–carbon nanostructures have been investigated by encapsulating the metal NPs particles in the carbon materials, such as core–shell and yolk–shell structures.^22–24^ During the catalytic process, the carbon shell functioned as a barrier to prevent the encapsulated nanoparticle from coalescence. Furthermore, the excellent chemical and thermal stability of carbon coatings are especially beneficial for catalytic applications.^25,26^ It has been shown that various carbon spheres or hollow carbon shells as isolated nanoreactors are employed to restrict the growth of active metal nanoparticles, making the catalyst more stable.^27^ However, such systems still have some drawbacks. As the synthesis of the metal nanoparticles incorporated inside carbon spheres usually involves sequential coating, the size of the metal NPs is usually large. Such a challenge leads to a relatively low distribution density of the metal NPs and a low mass utilization of the metal catalysts. On the other hand, when core–shell and yolk–shell composites are fabricated by the reported methods, additional workup procedures, such as pretreatment of the templates with metal particles, surface coating and selective etching strategy, are indispensable.^28,29^ Therefore, a more convenient approach for preparation of carbon–metal catalyst with controllable metal size, high loading, and excellent dispersion stability will be appreciated.^30^

Here, we report a simple and unique method for obtaining a carbon–metal composite catalyst, that is, Pt nanoparticles incorporated in mesoporous carbon nanospheres. Firstly, Pt/resorcinol-formaldehyde resin (RF) polymer nanocomposite was synthesized by a modified one-step strategy.^31^ The nanocomposite was further transformed to Pt/carbon nanostructures (Pt/C) by calcination in an inert Ar atmosphere. We demonstrate that the morphology and Pt content of the Pt/carbon composites can be tuned by manipulating the relative ratio of Pt precursor and RF. Taking the reduction of 4-nitrophenol (4-NP) as the example, we also show that Pt/C nanostructures are promising in catalytic applications. The nanocomposites showed high catalytic activity and stability. By stabilizing densely dispersed ultrafine Pt NPs within the mesoporous carbon spheres (denoted as 2Pt/C), the catalytic activity of the Pt/C can be optimized. The Pt/C composite with a loading of 6.6% preserved the excellent catalytic activity and stability.

## Experimental

### Chemicals

Tetraethyl orthosilicate (TEOS, 99%), hydrogen hexachloroplatinate(iv) (H_2_PtCl_6_·6H_2_O, 99.9%), resorcinol, ammonium aqueous solution (NH_3_·H_2_O, 28%), and 4-nitrophenol, were purchased from Sigma-Aldrich. Ethanol and formaldehyde (37 wt%) were obtained from Fisher Scientific. All chemicals were used as received without further purification. De-ionized water was used throughout the experiments.

### Synthesis

Pt/RF resin composites were prepared through a modified sol–gel method.^31^ Typically, a mixture containing deionized water (20 mL), H_2_PtCl_6_·6H_2_O (4.6–18.4 μmol), resorcinol (230–460 μmol), and formaldehyde solution (0.07 mL) was mixed in a three-necked flask under vigorous magnetic stirring. The solution was then heated to boiling and kept for 25 min, aqueous ammonia solution (28%, 0.07 mL) was rapidly injected into the mixture. After stirring at 100 °C for 20 min, a solid precipitate was centrifuged out, washed with ethanol three times and re-dispersed in ethanol (20 mL) to give the Pt/RF sample.

### Calcination

The obtained Pt@RF resin composites were dried under vacuum heated under an Ar atmosphere to 800 °C at a heating rate of 5 °C min^−1^, maintained at 800 °C for 2 h, and cooled to room temperature.

### Characterization

The sample morphology was characterized by using a transmission electron microscopy (TEM, Tecnai12). Thermogravimetric analysis (TGA) data was collected using a Seiko SSC 5200 TG/DTA thermal analysis apparatus in the temperature range of 30–800 °C at a rate of 10 °C min^−1^. The surface area of the Pt/carbon (Pt/C) composites was measured with the Brunauer–Emmett–Teller (BET) method by using a nitrogen sorption instrument (Quantachrome NOVA 4200e). The pore size distribution plot was obtained by the Barrett–Joyner–Halenda (BJH) method.

### Catalytic reduction of 4-nitrophenol

The reduction of 4-NP by H_2_ were chosen as model reactions to test the catalytic activity and stability of the Pt/C catalysts. Aqueous solutions of 4-NP (0.3 mL, 0.01 M) were added to deionized water (24 mL). After bubbling H_2_ for 10 min, the Pt/C catalyst particles (1 mL, 1 mg mL^−1^) were injected. The reaction progress was monitored using a UV-vis spectrophotometer (HR2000CG-UV-NIR, Ocean Optics). The concentration of the 4-NP in the reaction media was followed as a function of time by using the intensity of the 400 nm absorption peak in order to obtain the kinetic data. Stability tests were carried out under the same conditions.

## Results and discussion

**Fig. 1:**
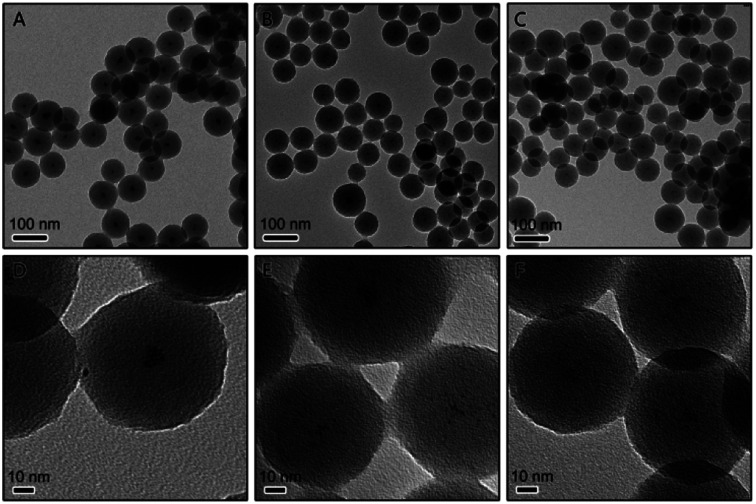
TEM image of Pt@RF composites prepared using (A and D) 230 μmol, (B and E) 345 μmol, and (C and F) 460 μmol of resorcinol.

The preparation of the Pt/carbon (Pt/C) composites involves the synthesis of Pt/resorcinol-formaldehyde (RF) resin composite and further carbonization. Firstly, the core–shell structured Pt/RF was synthesized by a sol–gel method. Resorcinol and formaldehyde were mixed with H_2_PtCl_6_ under vigorous magnetic stirring and then heated to boiling. In this process, formaldehyde acted as both a reductant of metal salts and a precursor of the resin. The H_2_PtCl_6_ can be reduced by formaldehyde to form Pt nanoparticles. Ammonia was then added to initiate the polymerization process to form a RF resin shell around the Pt nanoparticles. The monodisperse core–shell structured Pt/RF with a diameter of ∼80 nm can be obtained when 230 μmol R and 4.6 μmol H_2_PtCl_6_ were used (denote as 1Pt/RF). In high magnification images of each spherical particle (in Fig. 1D), it can be easily noticed that the core consist of aggregations of very small Pt NPs. As known, smaller size and better dispersity of Pt could increase the active surface area, which are beneficial for catalysis. In this system, R probably served as a surfactant that could stabilize the as-prepared Pt nanoparticles, thus we can assume that higher concentration of R probably can improve the dispersity of Pt particles in the porous carbon sphere. Therefore, the concentration of R was increased to 345 and 460 μmol, respectively. As shown in Fig. 1B, the obtained Pt/RF by adding 345 μmol R exhibited a similar size compared with 1Pt/RF. In the higher magnification image, it is observed that the Pt particles were less aggregated compared to 1Pt/RF. However, monodispersed Pt could not be obtained even the concentration of R was increase to 460 μmol, as shown in Fig. 1C and F.

**Fig. 2:**
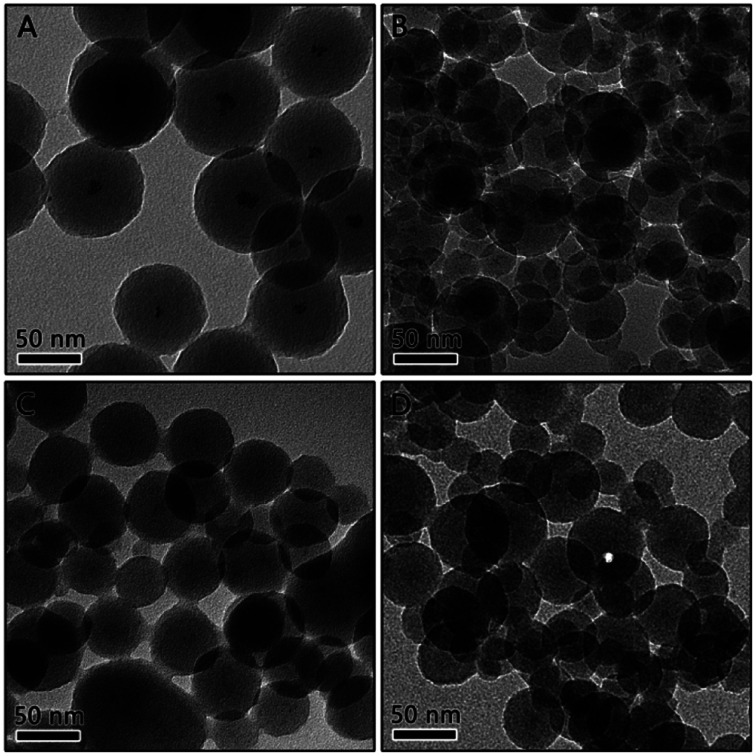
TEM image of Pt/RF composites prepared using (A) 4.6 μmol, (B) 9.2 μmol, (C) 13.8 μmol, and (D) 18.4 μmol of H_2_PtCl_6_.

To improve the dispersibility of Pt NPs, the concentration of H_2_PtCl_6_ was increased to provide more sites for the nucleation of Pt NPs. Fig. 2 shows the Pt/RF composites prepared by adding various amounts of Pt precursor when 230 μmol R was added. When the concentration of Pt precursor increased to 9.2 μmol, as expected, quite small Pt particles was well-dispersed in the RF sphere, as shown in Fig. 2B, confirming that the distribution of the Pt nanoparticles in the RF sphere can be adjusted by tuning the amount of the Pt precursor. Therefore, the concentration of Pt was further increased to 13.8 and 18.4 μmol, the resultant materials are denoted as 3Pt/RF and 4Pt/RF, respectively. As the Pt content increases, the Pt nanoparticles were dispersed homogenously as small and uniform dark spots over the entire RF spheres, as shown in Fig. 2C and D. However, the size of the spherical RF/Pt is not very uniform, with some aggregations.

**Fig. 3:**
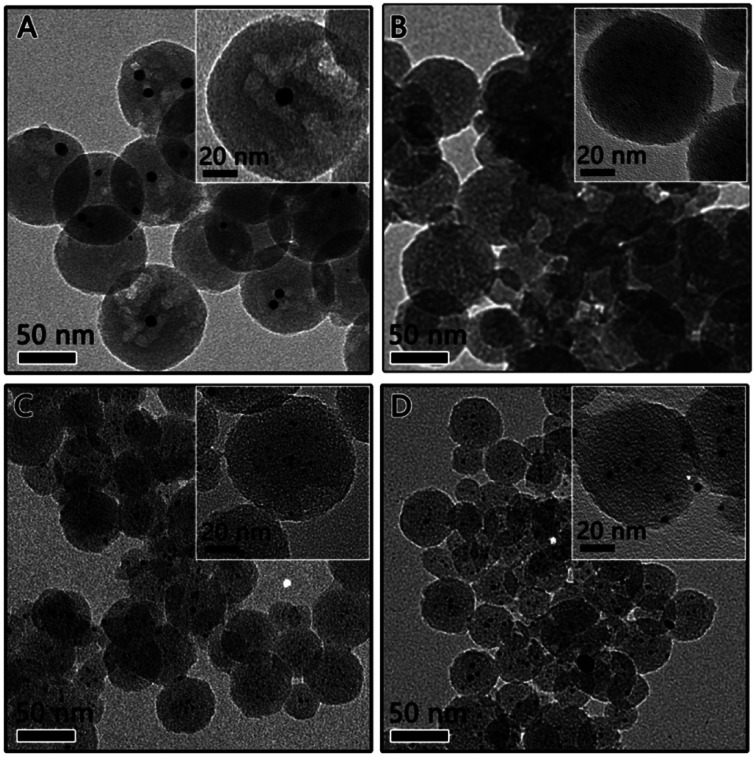
TEM image of (A) 1Pt/C, (B) 2Pt/C, (C) 3Pt/C, and (D) 4Pt/C composites.

The above mentioned RF/Pt nanostructures were then carbonized at high temperature (800 °C) under an inert Ar atmosphere to convert the RF polymer into carbon. The transmission electron microscopy (TEM) characterization was carried out to determine the particle size and distribution of the Pt catalyst. As expected, the RF polymer resin was carbonized, producing a carbon sphere with reduced size and porous structure (as shown in Fig. 3). The TEM image of 1Pt/RF after carbonization indicates that it was mesoporous carbon spheres, each of which encapsulates several Pt nanoparticles (denoted as 1Pt/C, Fig. 3A). Pt nanoparticles inside the carbon sphere were measured to estimate a particle size of *ca.* 8–9 nm from the high resolution TEM image (inset image in Fig. 3A). As the concentration of Pt precursor increased to 9.2 μmol (denoted as 2Pt/C), the close observation (inset image in Fig. 3B) confirmed the well-dispersed Pt particles (∼1–2 nm) and highly mesoporous nature of the carbon sphere. Larger Pt particles were formed in the carbon spheres in 3Pt/C and 4Pt/C, as shown in Fig. 3C and D. It is clearly seen in Fig. 3C that all the carbon nanospheres are uniformly decorated by ∼4 nm sized Pt particles, with very few aggregations. For the 4Pt/C, the Pt particles were ∼5 nm, some aggregates of Pt can be found. This can be explained by that Pt NPs grow to bigger particles inside the carbon sphere with the increasing of Pt density.

The metal contents in the nanocomposite were determined by thermal gravimetric analysis (TGA) in air. The weight loss *versus* temperature profiles are shown in Fig. S1.† The actual loading amount of Pt in the 1Pt/C, 2Pt/C, 3Pt/C and 4Pt/C samples were estimated by the above method to be about 5.8, 6.6, 7.3 and 10.3 wt%, respectively. It is also confirmed that the composition of Pt/C composite could be readily controlled by tuning the concentration of Pt precursor during the synthesis of the RF/Pt composite. The chemical composition of Pt/C catalyst was further analyzed by X-ray photoelectron spectra (XPS). An elemental survey of XPS indicates the presence of C, O, and Pt elements in Pt/C catalyst (Fig. S2, ESI†). The binding energies of Pt 4f_5/2_ and Pt 4f_7/2_ peaks are 74.6 and 71.4 eV, respectively.

**Fig. 4:**
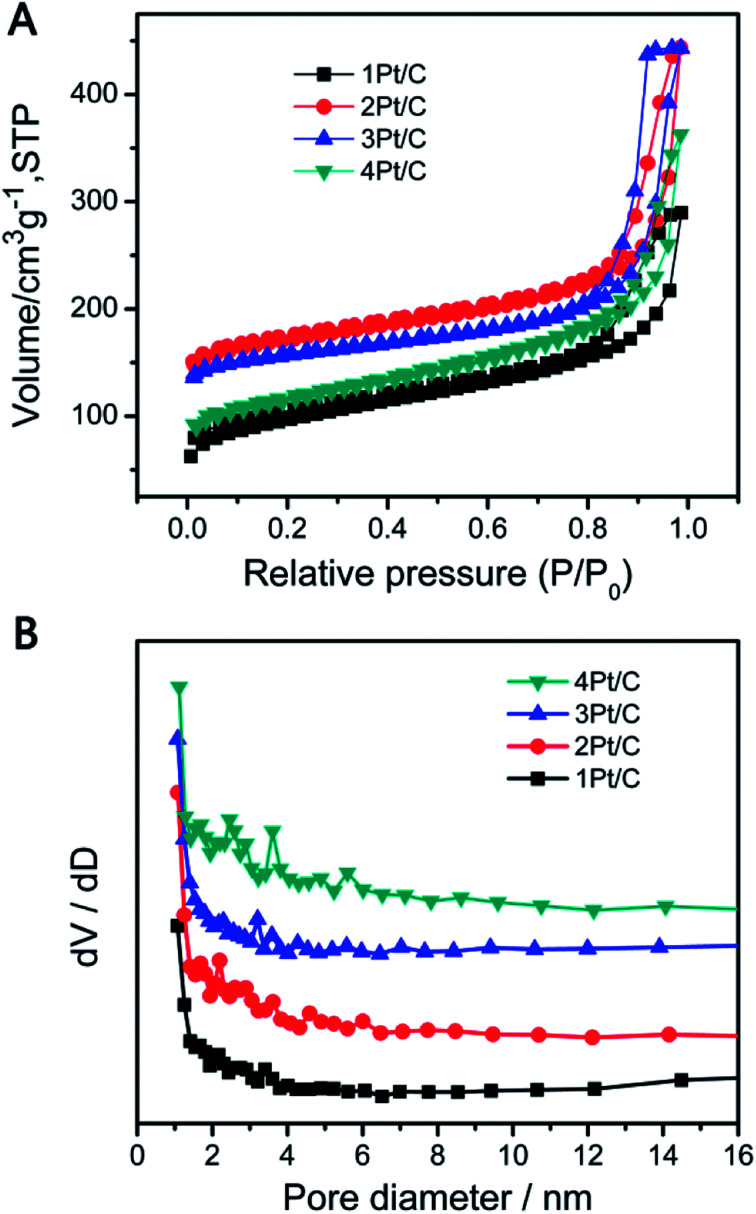
Nitrogen adsorption/desorption isotherms (A) and BJH pore-size distributions (B) of various Pt/C composites.

The surface area and pore size distribution data confirming the porous nature of our material. Fig. 4 shows the N_2_ adsorption–desorption isotherms and the corresponding pore size distribution for each sample, which can be classified as a type IV isotherm. The 1Pt/C composite exhibits a specific surface area of 401 m^2^ g^−1^, which is mainly attributable to the presence of the mesopores within the range of 2–4 nm (Fig. 4B). Structural parameters for the various Pt/C composite from the N_2_ adsorption–desorption isotherms are summarized in Table S1.† The surface area of the 2Pt/C, 3Pt/C and 4Pt/C are 600, 550, and 427 m^2^ g^−1^, respectively. It is clear that the 2Pt/C exhibits much larger BET surface area than the 1Pt/C, implying that the dispersed Pt particle is a potential catalyst for the carbonization of RF, resulting a mesoporous structure. However, the surface area decreased with further increase of Pt content, especially for 4Pt/C. The BET surface area of 4Pt/C sample was found to be 427 m^2^ g^−1^. This is probably because Pt has a high density in the 4Pt/C sample (10.3% as estimated from TGA analysis).

**Fig. 5:**
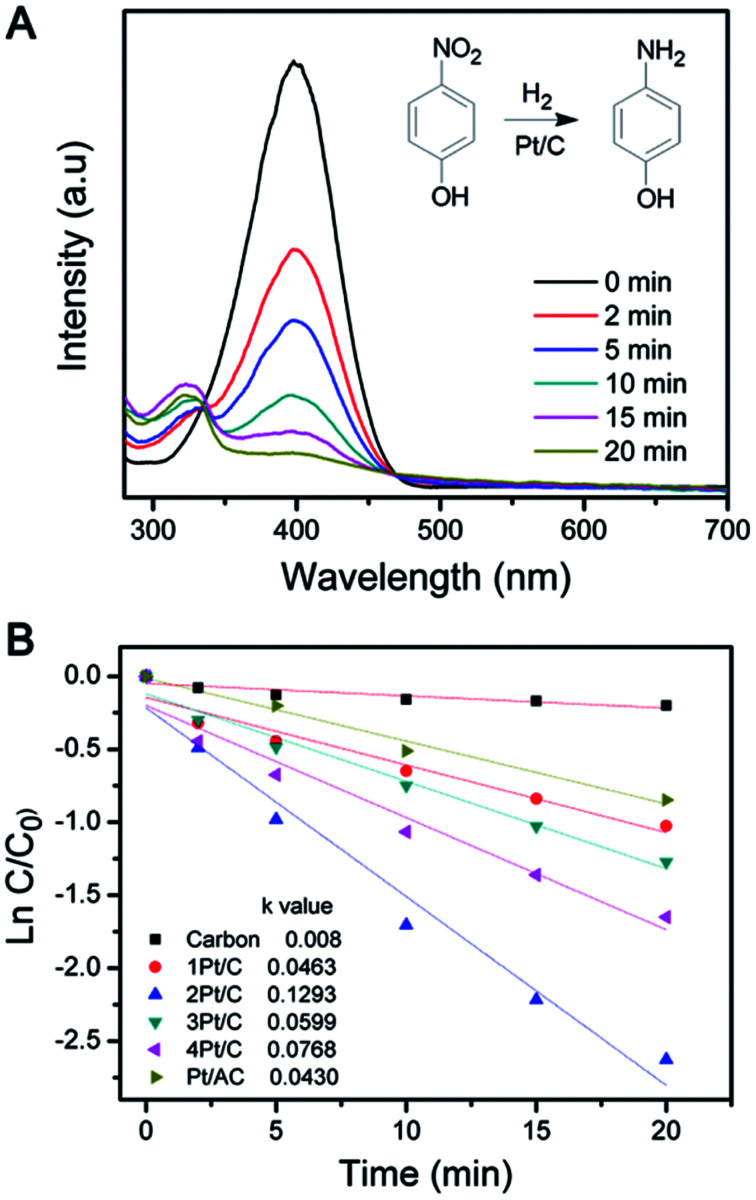
(A) UV-vis absorption spectra changes showing reduction of 4-NP using the 2Pt/C catalyst and (B) semilogarithmic plot *versus* time in the presence of different Pt/C catalysts.

A high active surface area of catalyst is usually beneficial for enhancing the catalytic activity.^32,33^ The reduction of 4-NP in the presence of H_2_ were used as model reactions to evaluate the catalytic performance of the Pt/C composite.^34^Fig. 5A shows the typical change of UV-vis absorption of the reaction mixture when catalyzed by Pt/C catalyst. The reduction reaction did not proceed with the presence of the carbon sphere (Fig. 5B). The weak decrease of the 4-NP concentration probably due to the adsorption by carbon sphere. However, the reaction was significantly improved when Pt/C catalysts were employed, which was evidenced by the decrease of the characteristic peak at 400 nm. The catalytic performance of the different catalysts was compared by fixing the Pt amount, which was determined by monitoring the intensity change of the 400 nm peak *versus* time, as summarized in Fig. 5A. The data were also plotted in semilogarithmic form in order to calculate the first-order reaction rate constants (*k*). As shown in Fig. 5B, 2Pt/C showed relatively higher *k* values (0.1293 min^−1^) than 1Pt/C (0.0463 min^−1^). This dramatic enhancement is likely from the high active surface area of well-dispersed Pt NPs and also the reduced diffusion resistance due to the high porosity of carbon composite. Since the 1Pt/C show a core–shell structure, the small active surface area of Pt resulting low catalytic activity. The relative catalytic activity of the catalysts for 4-NP reduction follows the order: 2Pt/C > 4Pt/C > 3Pt/C > 1Pt/C. Due to the aggregation of the Pt particles, the rate constant *k* of 3Pt/C and 4Pt/C decreased significantly to 0.0599 min^−1^ and 0.0768 min^−1^, respectively. It is apparent that the catalytic activity decreases when the Pt loading is higher than 6.6%. On the other hand, 4Pt/C and 3Pt/C still exhibit higher activity than 1Pt/C. For comparison, Pt supported on activated carbon (Pt/AC) was also made by using conventional synthetic route,^35^ where Pt nanoparticles were only simply deposited onto activated carbon substrate through the reduction of H_2_PtCl_6_ with NaBH_4_. The catalytic activity was evaluated in the same conditions as described above. It is worth to note that all the as-prepared Pt/C composite exhibit higher activity than the Pt/AC. The 2Pt/C composite, with a Pt loading of 6.6%, showed highest catalytic activity. This probably was attributed to the small size and uniform distribution of Pt NPs.

**Fig. 6:**
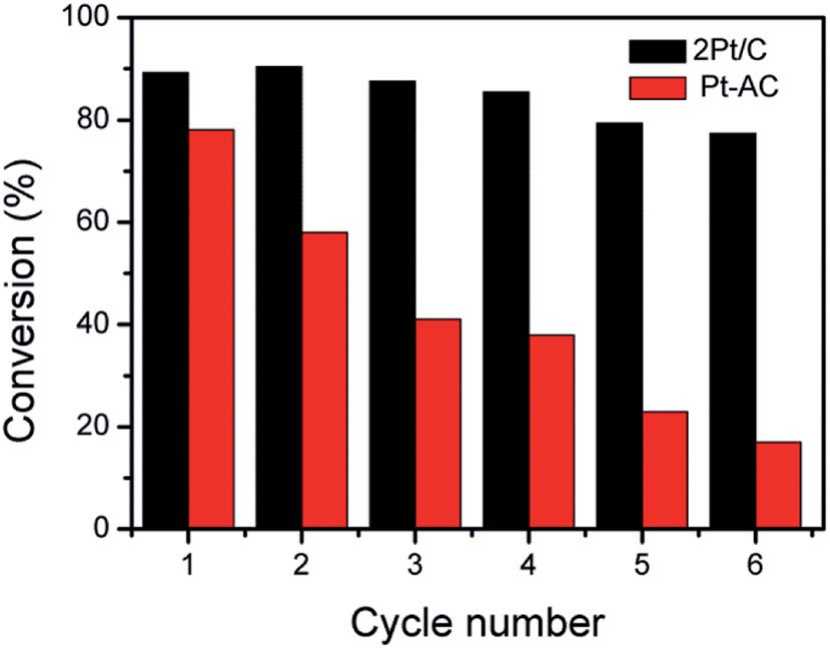
Conversions of 4-nitrophenol in several cycles catalyzed by 2Pt/C spheres and Pt/AC.

Stability and reusability are important concerns for heterogeneous catalysts.^36,37^ The 2Pt/C composite were therefore used in the reduction of 4-NP over several times to evaluate the reusability. After each reaction, catalysts were separated from solution by centrifugation, rinsed with deionized (DI) water, and redispersed into DI water for the next cycle of catalysis. The 2Pt/C catalyst showed no severe decay in catalytic activities in repeated experiments, as shown in Fig. 6. After six successive reaction cycles, 2Pt/C spheres were still quite active with the conversion efficiency of 77% within 5 min of reaction time. The slight decrease (from 89% to 77%) in catalytic efficiencies may be ascribed to the minor loss of catalysts during recycling. For comparison, the reduction reaction with Pt/AC catalyst was carried out under the same conditions. The conversion rate of 4-NP dropped significantly from ∼78% to ∼17% after six cycles. Such poor stability can be ascribed to the detachment of tiny Pt NPs from the surface of carbon. The 2Pt/C preserved the excellent stability and reusability. Apparently, the presence of the porous carbon shell is sufficient for stabilizing the catalytic Pt nanoparticles by preventing their aggregation; at the same time, the shells are permeable enough so that catalytic surfaces remain accessible to the reactants and products.

## Conclusions

In summary, we have demonstrated the fabrication of Pt nanoparticles encapsulated in carbon nanospheres using a sol–gel synthesis and carbonization method. With densely distributed Pt NPs, these spheres were found to have high surface area and well-developed meso-porosity, leading to their availability as catalysts. The Pt/C composite exhibit enhanced catalytic performance for reduction reactions of 4-nitrophenol. The preparation technique can be extended to fabrications of other highly efficient and stable heterogeneous catalysts including various metal nanoparticles other than Pt, such as Au and Pd.

## Conflicts of interest

There are no conflicts to declare.

## Supplementary Material

RA-008-C8RA01453A-s001
