# Supplementary material for: Pt incorporated mesoporous carbon spheres: controllable structure with enhanced catalytic activity and stability
Source: RSC Adv. 2018 Apr 16;8(25):13964–9. doi: 10.1039/c8ra01453a (PMC9079855; doi:10.1039/c8ra01453a)
Supplement: RA-008-C8RA01453A-s001 [file RA-008-C8RA01453A-s001.pdf]

Electronic Supplementary Material (ESI) for RSC Advances.
This journal is © The Royal Society of Chemistry 2018

Pt Incorporated Mesoporous Carbon Spheres: Controllable Structure with Enhanced Catalytic Activity and Stability

Hongyan Liu, * Yaling Liu, Huimei Da, Ruo Yuan

Key Laboratory of Luminescent and Real-Time Analytical Chemistry, Ministry of Education, College of Chemistry and Chemical Engineering, Southwest University, Chongqing 400715, P. R. China

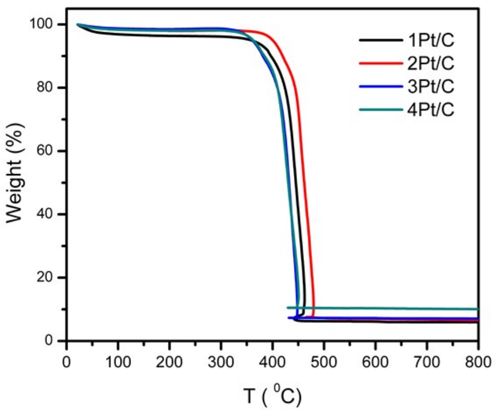

Fig. S1 TGA curves to determine Pt loading in the Pt/C composites

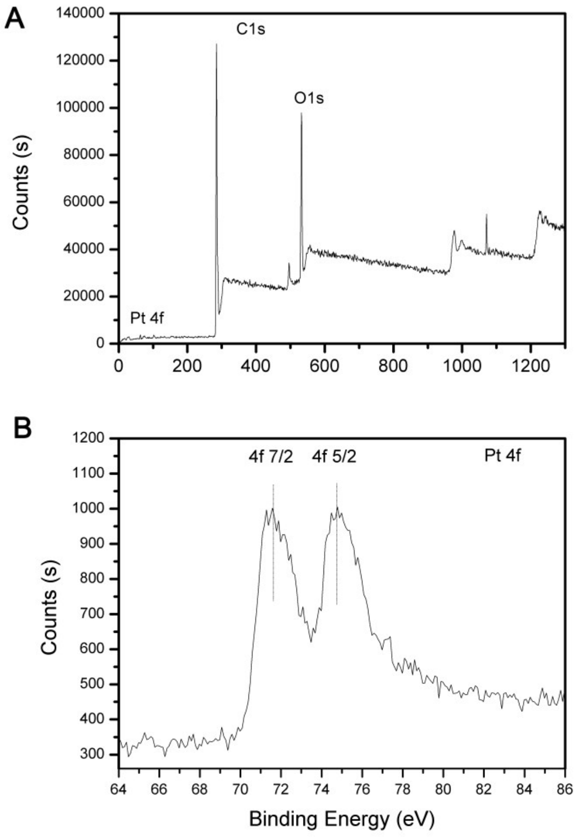

Fig. S2 XPS spectra of (A) 2Pt/C catalyst and (B) Pt 4f of 2Pt/C.

Table S1 Structural parameters for the various Pt/C composite from the N_2 adsorption–desorption isotherms.

Sample	BET Surface area(m^2/g)	Average pore size (nm)	Pore volume
1Pt/C	401	5.5	0.56
2Pt/C	600	4.6	0.68
3Pt/C	550	5.0	0.68
4Pt/C	427	5.3	0.71
